# Risk Perception and Risk-Taking Behaviour during Adolescence: The Influence of Personality and Gender

**DOI:** 10.1371/journal.pone.0153842

**Published:** 2016-04-21

**Authors:** Renate L. E. P. Reniers, Laura Murphy, Ashleigh Lin, Sandra Para Bartolomé, Stephen J. Wood

**Affiliations:** 1 University of Birmingham, School of Psychology, Birmingham, United Kingdom; 2 Telethon Kids Institute, The University of Western Australia, Perth, Australia; 3 Melbourne Neuropsychiatry Centre, University of Melbourne and Melbourne Health, Melbourne, Australia; Nathan Kline Institute and New York University School of Medicine, UNITED STATES

## Abstract

This study investigated the influence of personality characteristics and gender on adolescents’ perception of risk and their risk-taking behaviour. Male and female participants (157 females: 116 males, aged 13–20) completed self-report measures on risk perception, risk-taking and personality. Male participants perceived behaviours as less risky, reportedly took more risks, were less sensitive to negative outcomes and less socially anxious than female participants. Path analysis identified a model in which age, behavioural inhibition and impulsiveness directly influenced risk perception, while age, social anxiety, impulsiveness, sensitivity to reward, behavioural inhibition and risk perception itself were directly or indirectly associated with risk-taking behaviour. Age and behavioural inhibition had direct relationships with social anxiety, and reward sensitivity was associated with impulsiveness. The model was representative for the whole sample and male and female groups separately. The observed relationship between age and social anxiety and the influence this may have on risk-taking behaviour could be key for reducing adolescent risk-taking behaviour. Even though adolescents may understand the riskiness of their behaviour and estimate their vulnerability to risk at a similar level to adults, factors such as anxiety regarding social situations, sensitivity to reward and impulsiveness may exert their influence and make these individuals prone to taking risks. If these associations are proven causal, these factors are, and will continue to be, important targets in prevention and intervention efforts.

## Introduction

Taking risks encompasses behaviour that at the same time involves the chance of a beneficial outcome as well as possible negative or harmful consequences [1, 2]. Risk-taking behaviour increases during adolescence [3, 4], in association with heightened reactivity to emotions and a still immature ability to self-regulate [5, 6], making adolescence a period of high vulnerability to the negative consequences of risk-taking [7].

Recent research in adolescence [3, 8, 9] has mainly focussed on the modulation of risk-taking behaviour by emotional and social factors, such as the presence of peers. While adolescents spend a substantial amount of time with their peers, and are therefore likely to be influenced by them, there is more to the decision-making process including factors such as one’s genetic makeup [10–13], hormonal balance [14], psychological stress [15] and gender [16–18]. Likewise, factors such as maturity, responsibility, self-reliance, perspective [5, 19], anxiety and avoidance [20] should be considered. Individuals differ in their tendency to take risks and personality characteristics [5] including one’s sensitivity to reward (i.e. the positive response to the occurrence or anticipation of a reward, together with a desire, drive and willingness to pursue rewards [21]) and punishment (i.e.the susceptibility to present or future negative outcomes of behaviour [21]), impulsiveness (i.e. the predisposition toward rapid, unplanned reactions to stimuli without regard to the potential negative consequences of these behaviours [22]), sensation seeking, openness to experience and extraversion have commonly been linked to increased risk-taking behaviour (see for example [2, 23–29]). But what makes someone choose option A in a risky situation while another chooses option B? Which factors or personality characteristics that are specific to an individual may contribute to perception of risk, in other words the evaluated level of risk of a behaviour [30], and risk-taking behaviour, that is to say the frequency the behaviour is engaged in [30], during adolescence and what is their relative influence?

Research has shown that adolescents are sensitive to the immediate consequences of decisions [31], are strongly focussed on the anticipation of beneficial outcomes rather than associated costs [3, 32], and cope well with the unpredictability of a situation [33]. In addition to increased sensitivity to reward, adolescence has also been associated with increased impulsiveness and sensation seeking [2, 23–27]. It may be that this increased sensitivity to reward, which is associated with the rapid development of the brain’s reward system during adolescence, together with an immature ability to control one’s behaviour, which is associated with the more steadily development of the regulatory control system of the adolescent brain, contribute to the increase in risk-taking that is observed during this developmental period of one’s life [5, 34, 35]. Indeed, adolescents try new activities such as extreme sports, smoking and the consumption of alcohol and drugs, often without adequate assessment of the consequences or risks associated with their behaviour. Moreover, one’s levels of sensation seeking have been associated with strong approach and weak avoidance behaviours [36, 37], increased responsiveness to high-arousal stimuli [37] and reduced inhibitory control [36]. Even though they may not always behave accordingly [5, 38], adolescents are found able to refrain from taking risks but may require increased inhibitory control to do so [39]. This suggests that the way a risk is perceived in association with one’s personality is key for determining the occurrence of actual risk-taking behaviour.

The current study aimed to investigate the influence of personality characteristics on adolescents’ perception of risk as well as their risk-taking behaviour. In addition to age, the relative impact of sensitivity to reward and punishment, impulsiveness, and anxiety in social situations on risk perception and risk-taking behaviour was assessed. Older age, suggested to be associated with a better balance between one’s sensitivity to reward and regulatory control [5], and higher levels of anxiety in social situations were predicted to be associated with increased perception of risk and reduced risk-taking behaviour. As adolescents are found to be sensitive to the immediate consequences of decisions [31], increased sensitivity to punishment was also predicted to be associated with increased perception of risk and reduced risk-taking behaviour. Based on the available evidence of increased sensitivity to reward and reduced regulatory control during adolescence, sensitivity to reward and impulsiveness were predicted to relate to reduced perception of risk and increased risk-taking behaviour. Considering that adolescents are capable of reasoning about risk and estimating vulnerability to risk at a level similar to that of adults [5, 40], increased perception of risk was expected to be associated with reduced risk-taking behaviour. Males are reportedly more focussed on the potential beneficial outcome of a risky decision [3] and are more impulsive [41] than females, while females are found to be more sensitive to punishment and uncertainty [42, 43] and are more risk averse [16]. Thus additional analyses were conducted to examine the relative influence of the personality characteristics on risk perception and risk-taking behaviour for males and females separately.

## Materials and Methods

### Participants

A document describing the background and study requirements was sent to secondary schools who had previously indicated interest in collaborating with the University of Birmingham. Schools who expressed interest in taking part in the current study were provided with more detailed information and further arrangements regarding the testing were made. In return for the schools taking part in the research, members of the research team offered talks related to their research expertise, shared their experiences of studying psychology with the students and gave students more insight in the ins and outs of conducting research. Parents/legal guardians of the students gave written informed consent for their child to take part in the research prior to the testing day. Students themselves gave written assent on the testing day. Ethical approval was granted by the School of Psychology Ethics Board of the University of Birmingham.

356 students (204 females: 152 males corresponding to 57.3% and 42.7% of the sample) from 7 secondary schools in the West Midlands, United Kingdom, were recruited for this study. The only exclusion criterion was non-fluency in English. Socioeconomic status of the students was mixed with 42.4% of the students attending an independent (private) school and 57.6% attending a comprehensive state school. 273 students (70% of the total sample, 157 females: 116 males) had a maximum of 10% of any subscale measures missing. For these participants missing values were replaced with the mean of the person’s scores on that subscale [44], and their data with missing values imputed were included in further analysis. Data of the remaining 30% (n = 83) were excluded; they did not differ from the included sample. 75.5% of the final sample were born in the United Kingdom. Ethnicity was 46.9% white, 20.9% Asian-Indian, 11.0% Asian-Oriental, 8.1% Black/African-Caribbean, 7.7% mixed and 5.5% other ethnic background.

### Measures

The *Adolescent Risk-Taking Questionnaire (ARQ)* [45] is a two-part questionnaire that assesses adolescent risk-taking behaviours in addition to perceptions of the risk of these behaviours. The questionnaire contains 44 items that are rated on a 5-point Likert scale for judgment of riskiness (e.g. “Put a cross in the box corresponding to the word that best describes your opinion about how risky you think each situation or behaviour is: smoking”) and frequency of participation in the behaviours (e.g. “Put a cross in the box corresponding to the word that best describes your behaviour: smoking”). The ARQ has adequate internal reliability with Cronbach’s alphas ranging from 0.7 to 0.79 for the antisocial subscales and Cronbach’s alphas exceeding 0.8 for the other subscales for males, females, young adolescents and older adolescents [30]. For the current study, Cronbach’s alphas ranged from 0.40 to 0.83 with poor internal consistency for items referring to thrill-seeking and reckless risk behaviour and good internal consistency for items referring to rebellious risk behaviour.

The *Behavioral Inhibition System/Behavioral Activation System (BIS/BAS) scales* [21] assess motivational systems that underlie behaviour and affect. The behavioural inhibition system (BIS) is sensitive to punishment, non-reward, and novelty, and is associated with inhibition of behaviour that may result in negative outcomes (e.g. “I worry about making mistakes”). The behavioural activation system (BAS) is sensitive to reward, non-punishment and escape from punishment. There are three BAS subscales: drive (e.g. “I go out of my way to get things I want”), fun-seeking (e.g. “I'm always willing to try something new if I think it will be fun”) and reward responsiveness (e.g. “When I get something I want, I feel excited and energized”) which, for the purpose of this study, have been summed to get a total score that is associated with behaviour aimed to obtain reward. The questionnaire consists of 24 items, rated on a 4-point Likert scale, and has adequate internal reliability with Cronbach’s alphas ranging from 0.68 to 0.88 on the subscales for participants in the US, UK and Italy [46] and 0.55 to 0.73 for the current study. The BIS/BAS scales also have adequate convergent and discriminant validity [21].

The *Barratt Impulsiveness Scale (BIS-11)* [47] is the most widely used self-report measure of impulsivity [48] and assesses impulsiveness in the form of attentional (e.g. “I have “racing” thoughts”), motor (e.g. “I do things without thinking”) and non-planning impulsivity (e.g. “I get easily bored when solving thought problems”). The measure consists of 30 items that are rated on a 4-point Likert scale and summed to obtain a total score for impulsiveness. The BIS-11 has adequate internal reliability with Cronbach’s alphas ranging from 0.79 to 0.83 for undergraduate students, substance-abuse patients, general psychiatric patients and prison inmates [47] and 0.52 to 0.68 for the current study.

The *Social Interaction Anxiety Scale (SIAS) and the Social Phobia Scale (SPS)* [49] assess anxiety symptoms related to initiating and maintaining interactions with people in social situations as well as anxiety symptoms in relation to performance of various tasks while being observed by other people (e.g. SIAS “When mixing socially, I am uncomfortable”; e.g. SPS “I become nervous if I have to write in front of other people”). Both scales consist of 20 items, rated on a 5-point Likert scale, and are summed to obtain a score representing anxiety related to social situations. The scales have good to excellent internal reliability with Cronbach’s alphas of 0.9 for the SPS and 0.88 and 0.9 for the SIAS for undergraduate and community samples respectively [49] and 0.94 for the SPS and 0.92 for the SIAS in the current study.

### Procedure

Students completed the questionnaires in their classrooms. They were asked to answer the questions honestly and not discuss their answers with their friends while completing the measures. Questionnaire items that were difficult to understand were explained in a standardised way that was agreed between the researchers prior to the start of the study. Students were given a sheet that included contact details of organisations for advice on mental health. The duration of a typical testing session was 40–50 minutes.

### Statistical analysis

Data were analysed using IBM SPSS Statistics 21 and IBM SPSS Amos 21 for Windows (IBM Corp., Armonk, NY). Path analysis was employed to investigate the influence of personality characteristics on adolescents’ perception of risk and their risk-taking behaviour with plausibility of the postulated relationships being indicated by a good fitting model [50, 51]. Models were estimated using a maximum likelihood algorithm and model fit was judged using guidelines provided by Byrne (2001), Hu & Bentler (1999) and Kline (1998). Path coefficients and the amount of variance explained by the model (R^2^) were examined and the following goodness of fit measures are reported: the model χ², Root Mean Squared Error of Approximation (RMSEA) with its 90% confidence intervals, Bentler’s Comparative Fit Index (CFI), Tucker-Lewis Index (TLI), Standardised Root Mean Square Residual (SRMR) and Aikaike’s Information Criterion (AIC).

## Results

Descriptive information on participants’ performance on the self-report measures is presented in Table 1. Male and female participants did not significantly differ in age or self-reported levels of sensitivity to reward and impulsiveness. Males perceived behaviour as less risky (*Z* = -2.054, *p*<0.05 with an effect size [52, 53] of *r* = -0.12), took more risks (*Z* = 2.043, *p*<0.05, *r* = 0.12), were less sensitive to negative outcomes (present or future outcomes) (*Z* = -4.726, *p*<0.001, *r* = -0.29) and were less socially anxious (*Z* = -2.228, *p*<0.05, *r* = -0.13) than the female participants.

**Table 1:** Sample characteristics.

Measure	Median			Range		
	Total sample	Males	Females	Total sample	Males	Females
	n = 273	n = 116	n = 157	n = 273	n = 116	n = 157
**Age**	17.13	17.23	17.11	13.35–19.69	13.35–19.69	13.55–19.07
Risk perception*	48	47	49	17–86	17–77	24–86
Risk-taking behaviour*	18	20	18	2–47	3–47	2–47
Behavioural inhibition**	21	19	21	10–28	10–27	12–28
**Reward**	39	39	39	23–52	26–50	23–52
**Impulsiveness**	68	69	67	43–92	43–90	45–92
Social anxiety*	41	37	47	3–151	3–105	6–151

** Significant difference between genders at *p*<0.001

* Significant difference between genders at *p*<0.05

**Fig 1:**
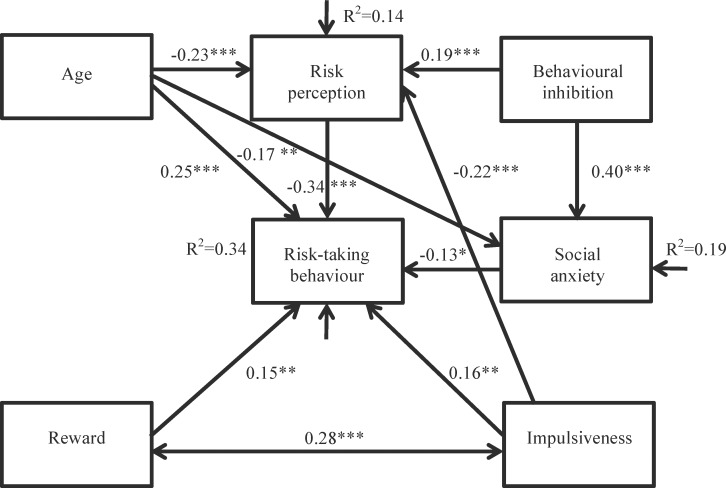
Path model exploring the relationship between risk perception, risk-taking behaviour, age, and personality characteristics. * *p*<0.05; ** *p<*0.01; *** *p*<0.001. Boxes represent observed variables. Long, solid arrows represent regressions. Short arrows represent residual error variances that indicate the variation left unexplained by the variables in the path model. Numbers indicate the standardised regression weights and R^2^ indicates the amount of variance explained by the model.

The first path model (model 1, S1 Fig) showed age, sensitivity to reward, behavioural inhibition, impulsiveness and anxiety in social situations as variables that influence both risk perception and risk-taking behaviour. Older age, sensitivity to punishment and higher levels of anxiety in social situations were predicted to be associated with increased perception of risk and reduced risk-taking behaviour. Sensitivity to reward and impulsiveness, on the other hand, were predicted to relate to reduced perception of risk and increased risk-taking behaviour. Risk perception was also depicted to influence risk-taking behaviour with perception of low risk being expected to increase risk-taking behaviour. Model 1 did not provide a good fit to the data (see Table 2) with relationships for both social anxiety and reward with risk perception, and behavioural inhibition with risk behaviour being redundant (*p*>0.05) and relationships for behavioural inhibition with social anxiety, and reward with impulsiveness being suggested. Consistent with the considerations that those who are more sensitive to punishment may be inclined to be more anxious in social situations, the model with this association demonstrated improved fit over the model with the association in the opposite direction. With evidence suggesting an interaction between reward sensitivity and behavioural control [5, 6, 54], the association between reward and impulsiveness was specified as one of covariance. The modified model (model 2, S2 Fig) with the recommended changes incorporated provided a reasonable fit to the data (see Table 2) but an additional association for age with social anxiety was suggested. The effect peers have on behaviour has been shown to decline between adolescence and adulthood [3, 8], making a decline in anxiety related to social situations with age likely. The model with age influencing social anxiety demonstrated improved fit over the model with the association specified in the opposite direction and is presented in model 3. Model 3 provided a good fit to the data with significant loadings for all factor indicators at *p*<0.05. Models 2 and 3 were nested and significant improvement in fit for the model with the additional path (model 3) was confirmed: Δχ^2^(1) = 9.074, *p*<0.005. Model 3 explained 14% of the variance in risk perception, 34% of the variance in risk-taking behaviour and 19% of the variance in social anxiety. These values and the standardised regression weights of model 3 are presented in Fig 1. A sensitivity analysis for non-normality with bootstrapping (Bollen-Stine bootstrap *p* = 0.202) confirmed good model fit. Standardised indirect and total effects predicting risk-taking behaviour are presented in Table 3, as well as the standard errors and 95% confidence intervals corresponding to each parameter.

**Table 2:** Goodness of fit tests and indices.

Model	Parameters	Goodness of fit measure					
	(estimated)	χ²(df), *p*	RMSEA (90% CI)	CFI	TLI	SRMR	AIC
**Model 1**	28 (18)	χ²(10) = 88.264, *p*<0.001	0.170 (0.138–0.203)	0.656	0.277	0.1045	124.264
**Model 2**	28 (17)	χ²(11) = 24.541, *p* = 0.011	0.067 (0.031–0.103)	0.940	0.886	0.0479	58.541
**Model 3**	28 (18)	χ²(10) = 15.467, *p* = 0.116	0.045 (0.000–0.086)	0.976	0.950	0.0371	51.467

df = degrees of freedom, RMSEA = Root Mean Squared Error of Approximation, CI = confidence interval, CFI = Bentler’s Comparative Fit Index, TLI = Tucker-Lewis Index, SRMR = Standardised Root Mean Square Residual and AIC = Aikaike’s Information Criterion

**Table 3:** Standardised indirect and total effects for predicting risk-taking behaviour.

Predictor	Mediator	Indirect effects				Total effects
			S.E.	95% C.I.		
				Lower	Upper	
Behavioural inhibition		-0.113				-0.113
	Risk perception		0.051	-0.253	-0.053	-0.336
	Social anxiety		0.051	-0.222	-0.022	-0.125
Impulsiveness		0.075				0.237
	Risk perception		0.021	0.031	0.111	-0.336
Age		0.098				0.346
	Risk perception		0.145	0.218	0.783	-0.336
	Social anxiety		0.071	0.016	0.291	-0.125

The measures correspond to the model presented in Fig 1. S.E., standard error; C.I., confidence interval.

Structural equation modelling allows comparison of parameter estimates and model structure in different groups [50, 55]. Additional analyses comparing the models for males and females to the corresponding model 3 with equality constraints resulted in Δχ²(20) = 23.240, n.s. Model 3 was therefore concluded to be representative for the whole sample as well as for both genders separately. As there is evidence to suggest that males are more focused on reward [3] and are more impulsive [41] than females, while females are found to be more sensitive to punishment [42, 43], the models for males and females were compared to the corresponding model 3 with equality constraints for these particular pathways: reward, impulsiveness and sensitivity to punishment with risk-taking and risk perception. None of the tests for invariance were statistically significant (Δχ²(11) = 14.973, n.s. for the comparison of the models for males and females to the corresponding model 3 with the path between reward and risk-taking constrained; Δχ²(11) = 16.138, n.s. for the comparison with the path between impulsiveness and risk-taking constrained; Δχ²(11) = 14.575, n.s. for the comparison with the path between impulsiveness and risk perception constrained; and Δχ²(11) = 14.884, n.s. for the comparison with the path between behavioural inhibition and risk perception constrained), suggesting that all factor loadings are equivalent across males and females.

In England, 7% of adolescents currently attend an independent school [56]. As in the current sample more than 40% of the students attended an independent school, an extended model (model 4) was generated including school type as a variable associated with risk-taking behaviour, risk perception and age. The latter path was bi-directional and included age as students attending an independent school were significantly older than students attending a comprehensive school (*Z* = -2.772, *p*<0.05, *r* = -0.17; independent school n = 122, median age 17.6, range 13.4–19.7; comprehensive school n = 151, median age 16.1, range 13.6–18.5). Model 3 had the lower AIC compared to model 4 (AIC_model3_ = 51.467 versus AIC_model4_ = 62.120), and was therefore concluded to be the better model [57] (S3 Fig and S1 Table for details on model 4).

## Discussion

The current study investigated the influence of personality characteristics and gender on adolescents’ perception of risk as well as their risk-taking behaviour. Male and female participants did not significantly differ in age or self-reported levels of sensitivity to reward and impulsiveness but males perceived behaviour as less risky, took more risks, were less sensitive to negative outcomes and less socially anxious than female participants. Path analysis identified a model in which age, behavioural inhibition and impulsiveness were directly associated with risk perception, while age, social anxiety, impulsiveness, sensitivity to reward, and risk perception itself were directly associated with risk-taking behaviour. Furthermore, age and behavioural inhibition had direct relationships with social anxiety and reward sensitivity was associated with impulsiveness. The model provided good fit and was representative for the whole sample as well as male and female groups separately.

Contrary to prediction, older age was associated with an increase in risk-taking behaviour in our sample. Research suggests that the highest sensitivity to reward lies around 12–13 years of age [6] and that behavioural control improves at a more steadily pace during adolescence [5], leading us to the prediction that risk-taking in our sample would decrease with age. However, many risk-taking behaviours, such as dangerous driving and excessive alcohol use, have been shown to emerge, increase and peak between the ages of 12 and 18 [2]. Perhaps the freedom that comes with age (e.g. being allowed to drive a car, drink alcohol) plays a more prominent role in explaining the increase in risk-taking than anticipated. This argues for the careful consideration of age in this context, in addition to the developmental context, in its impact on adolescent risk-taking behaviour.

Social anxiety was influenced by age with older age in this sample indicating reduced concern about social situations and perhaps reduced sensitivity to external social factors. Importantly, increased social anxiety was associated with reduced risk-taking behaviour but not risk perception. This may be where adolescents differ from adults and this has therefore important implications for reducing risk-taking behaviour in adolescence. Even though adolescents may understand the riskiness of their behaviour and estimate their vulnerability to risk at a similar level to adults [5, 40], other factors such as anxiety regarding social situations, but also sensitivity to reward and impulsiveness, may exert their influence and make these individuals prone to taking risks. If these associations are proven causal, these factors are [58–60], and will continue to be [61], important targets in prevention and intervention efforts to reduce risk-taking behaviour during adolescence.

Consistent with previous research [42, 43], males reported less inhibition of behaviour that has potential negative outcomes than their female counterparts. As expected, increased behavioural inhibition was associated with increased risk perception but there was no direct relationship with risk-taking behaviour. This association was indirect, mainly via social anxiety. This suggests that one’s concern with both interactions in social situations and being observed by others reduces risk-taking behaviour, particularly when negative consequences are obvious or likely. This is consistent with recent findings of increased as well as reduced risk-taking behaviour when in the presence of peers [62].

Male participants perceived behaviour as less risky and took more risks than females. This is consistent with previous research showing females to be more risk averse than males [16]. Males may be more focused on the potential benefits than associated costs than females [3] but this was not translated in increased sensitivity to reward for males compared to females in the current study. It may rather be the perception of conducts to be less risky that fuelled male participants to take more risks than their female counterparts. Indeed, risk perception was strongly associated with risk-taking behaviour in model 3 with increased perception of risk reducing risk-taking behaviour.

Consistent with previous findings [63], males were less socially anxious than females suggesting less concern with the social situation they are acting in. Importantly, while male and female participants scored significantly different on measures of risk perception, risk-taking behaviour, behavioural inhibition and social anxiety, the model showing the relationships between these characteristics and behaviours did not differ between genders. Increased perception of risk and increased social anxiety reduced risk-taking behaviour, regardless of gender, and these factors were influenced by inhibition of behaviour with likely negative consequences.

Contrary to expectation [41], males were as impulsive as female participants and both genders were equally sensitive to potential rewards. Both factors showed a positive association with risk-taking behaviour which is consistent with previous research [24, 26, 27]. Interestingly, only impulsiveness showed a direct relationship with risk perception. Acting before thinking, not considering the future consequences of actions, and diminished attention reduce risk perception and increase risk-taking behaviour during adolescence. Model 3 suggested an important role for the interplay between sensitivity to reward and impulsiveness in association with increased risk-taking behaviour, a finding that is widely supported [5, 34, 35].

Importantly, the study is cross-sectional and as such relationships between variables can be identified but directionality is based on assumption. In order to test the predictive properties of variables and the therewith associated mediation, a longitudinal design should be employed. Older age in this study was associated with reduced risk perception and increased risk-taking behaviour. As risk-taking peaks in adolescence and reduces with maturation [5], the median age of the current sample (17 years) may suggest that the sample is at or close to the peak age for risk-taking behaviour. Notably, the current study presents findings of a cross sectional sample that shows increased risk-taking behaviour in those with older age. This does not mean, however, that if this sample was reassessed in a few years’ time, this association would have reversed and those with older age would show reduced risk-taking behaviour. Longitudinal studies will be needed to confirm the proposed quadratic relationship between age and risk-taking behaviour, taking into account other factors such as personality, as is done in the current study. Similarly, whether the relative influence of age on risk perception and social anxiety comprises the same quadratic relationship as has been suggested for risk-taking behaviour [34, 64, 65] remains a topic of great interest for future research.

The current study limited itself to the assessment of personality characteristics on risk perception and risk-taking behaviour. This does, however, not mean that the importance of other factors such as genetics, including one’s genetic makeup and the level of riskiness displayed by first degree relatives, hormonal balance, psychological stress, emotion and peer pressure should be ignored. Clearly, follow-up work would do well to be larger and cover multiple domains together with more detailed information regarding demographics of the sample. Future research should, for example, aim to combine assessment of state and trait factors to increase understanding of the interplay between these factors. Ideally, this should be done at different periods of development. Although a large sample spreading across the ages of 13 to 20 years old was obtained, the generalisability of the findings with regards to developmental processes is limited. Nevertheless, they present a model in which personality traits and age affect risk perception and risk-taking behaviour. Risk perception and risk-taking behaviour may change over time and are likely to vary with age, and the same has been shown for personality traits [66]. The current findings therefore suggest that their influence on risk perception and risk-taking behaviour should be considered when investigating the developmental processes that may influence risk perception and risk-taking behaviour during adolescence. Notably, the current study did not include a measure of mental age. This would have provided valuable insight into the relationship between brain maturation, risk perception and risk-taking behaviour. It needs noting that the current study recruited participants who are attending school while it is likely that a disproportionate number of strong risk-takers may be truants or may have dropped out of school. Whilst the percentage of participants in the current study attending an independent school is not representative for the national average (just over 40% versus 7%), the model without school type was shown to be the better one. This suggests that the current findings may be generalisable to the national adolescent population; however, replication in a sample with a more representative distribution is needed to confirm this.

## Conclusions

The current study investigated the influence of personality characteristics and gender on adolescents’ risk perception and risk-taking behaviour. While gender differences in risk perception, risk-taking behaviour, behavioural inhibition and social anxiety were observed, the same relationships between traits were shown for both genders. Age, behavioural inhibition and impulsiveness were associated with risk perception and risk-taking behaviour was directly associated with risk perception, age, social anxiety, impulsiveness and sensitivity to reward, and indirectly with behavioural inhibition. Age and behavioural inhibition also exerted direct influence on social anxiety, and reward sensitivity covaried with impulsiveness. The observed relationships of age and behavioural inhibition with social anxiety in its association with risk-taking behaviour may be key, particularly during adolescence. If future research is able to demonstrate causality in this association, this would urge for consideration in prevention and intervention efforts aimed at reducing risk-taking behaviour in this challenging age group. First attempts have been promising [59, 60] and an increased understanding of the mechanisms underlying individual differences in risk-taking behaviour (e.g. reward sensitivity, impulsiveness and social anxiety), as well as the vulnerabilities that occur during adolescence (i.e. the heightened reactivity to reward coupled with a still immature ability to self-regulate), may suggest ways in which prevention and intervention programs can be designed and administered to be sensitive to both individual differences and developmental timing [61]. Together, the relationships observed in the current study increase our understanding of risk perception and risk-taking behaviour in an adolescent sample and efforts should be made to investigate these personality characteristics within varying contexts and in a longitudinal setup to examine their relative influence in the interplay between state and trait factors within the developmental context.

## Supporting Information

S1 Fig Path model exploring the relationship between risk perception, risk-taking behaviour, age, school type, and personality characteristics. ^ *p*>0.05; * *p*<0.05; ** *p<*0.01; *** *p*<0.001. Boxes represent observed variables. Long, solid arrows represent regressions. Short arrows represent residual error variances that indicate the variation left unexplained by the variables in the path model. Numbers indicate the standardised regression weights and R^2^ indicates the amount of variance explained by the model.
(TIFF)

S2 Fig Path model exploring the relationship between risk perception, risk-taking behaviour, age, school type, and personality characteristics. * *p*<0.05; ** *p*<0.01; *** *p*<0.001. Boxes represent observed variables. Long, solid arrows represent regressions. Short arrows represent residual error variances that indicate the variation left unexplained by the variables in the path model. Numbers indicate the standardised regression weights and R^2^ indicates the amount of variance explained by the model.
(TIFF)

S3 Fig Path model exploring the relationship between risk perception, risk-taking behaviour, age, school type, and personality characteristics. * *p*<0.05; ** *p*<0.01; *** *p*<0.001. Boxes represent observed variables. Long, solid arrows represent regressions. Short arrows represent residual error variances that indicate the variation left unexplained by the variables in the path model. Numbers indicate the standardised regression weights and R^2^ indicates the amount of variance explained by the model.
(TIFF)

S1 Table Goodness of fit tests and indices. df = degrees of freedom, RMSEA = Root Mean Squared Error of Approximation, CI = confidence interval, CFI = Bentler’s Comparative Fit Index, TLI = Tucker-Lewis Index, SRMR = Standardised Root Mean Square Residual and AIC = Aikaike’s Information Criterion
(DOCX)
