# Supplementary figures and images for: Risk Perception and Risk-Taking Behaviour during Adolescence: The Influence of Personality and Gender
Source: PLoS One. 2016 Apr 21;11(4):e0153842. doi: 10.1371/journal.pone.0153842 (PMC4839773; doi:10.1371/journal.pone.0153842)

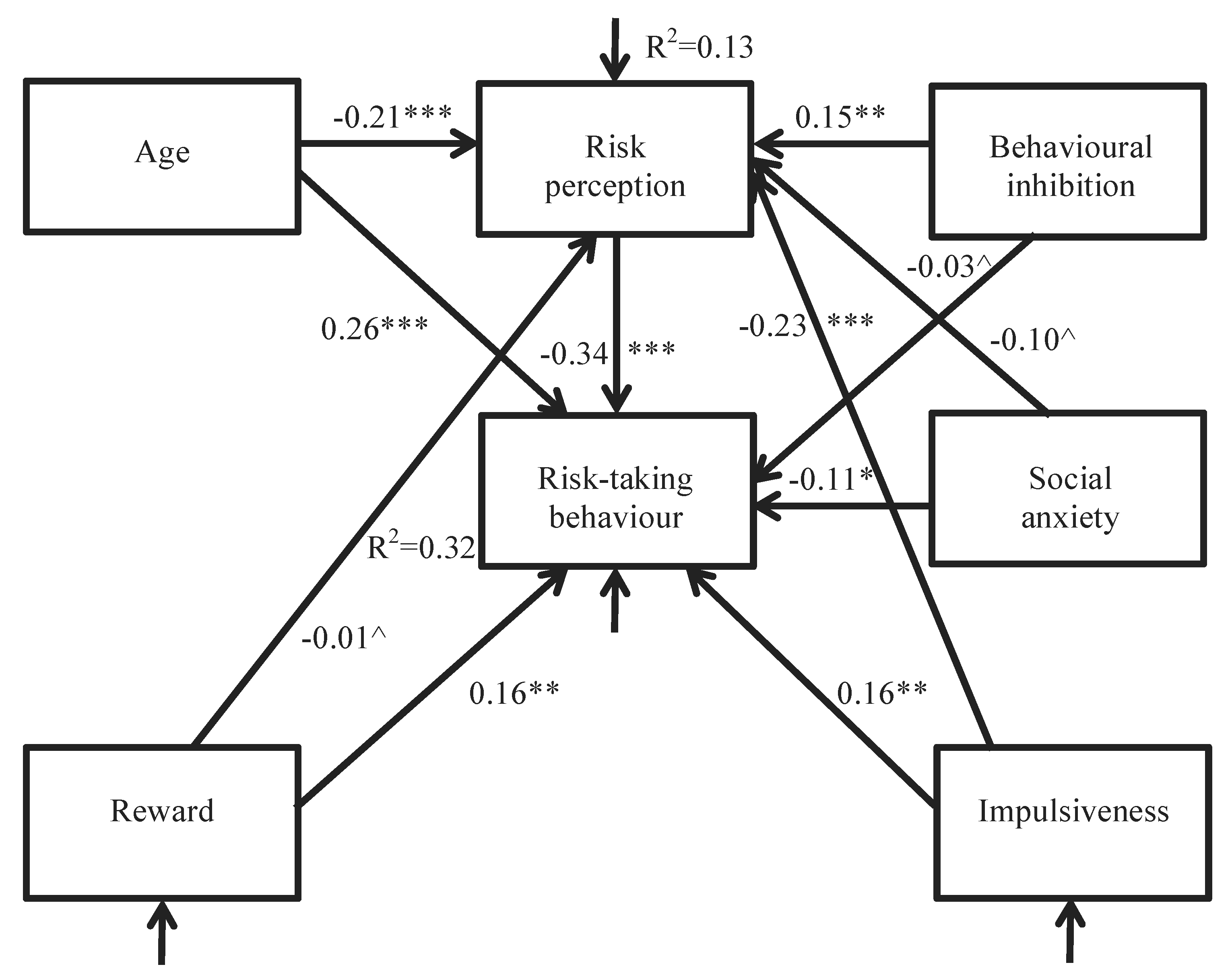

Supplement: S1 Fig — ^ p>0.05; * p<0.05; ** p<0.01; *** p<0.001. Boxes represent observed variables. Long, solid arrows represent regressions. Short arrows represent residual error variances that indicate the variation left unexplained by the variables in the path model. Numbers indicate the standardised regression weights and R2 indicates the amount of variance explained by the model. (TIFF) [file pone.0153842.s001.tiff]

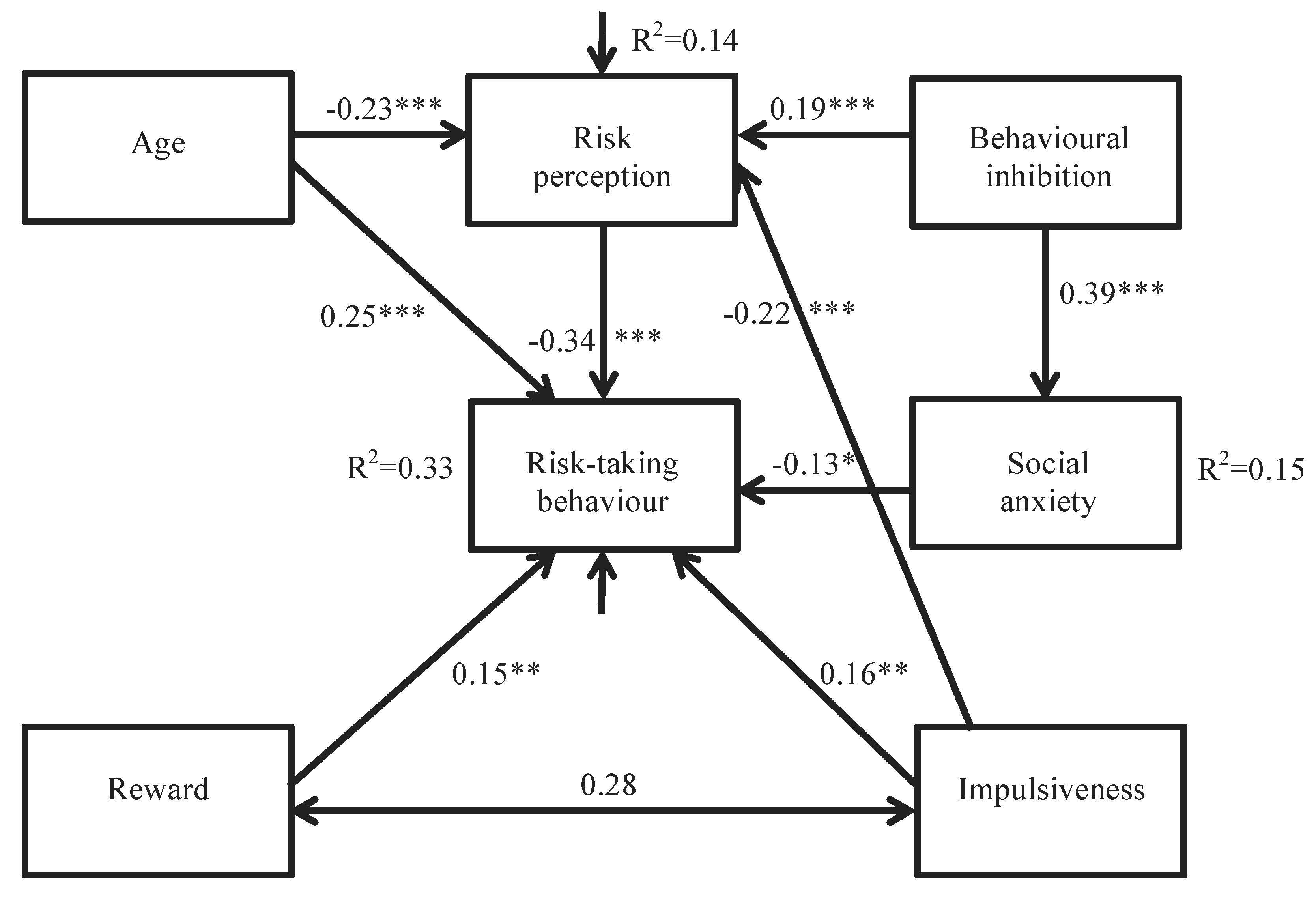

Supplement: S2 Fig — * p<0.05; ** p<0.01; *** p<0.001. Boxes represent observed variables. Long, solid arrows represent regressions. Short arrows represent residual error variances that indicate the variation left unexplained by the variables in the path model. Numbers indicate the standardised regression weights and R2 indicates the amount of variance explained by the model. (TIFF) [file pone.0153842.s002.tiff]

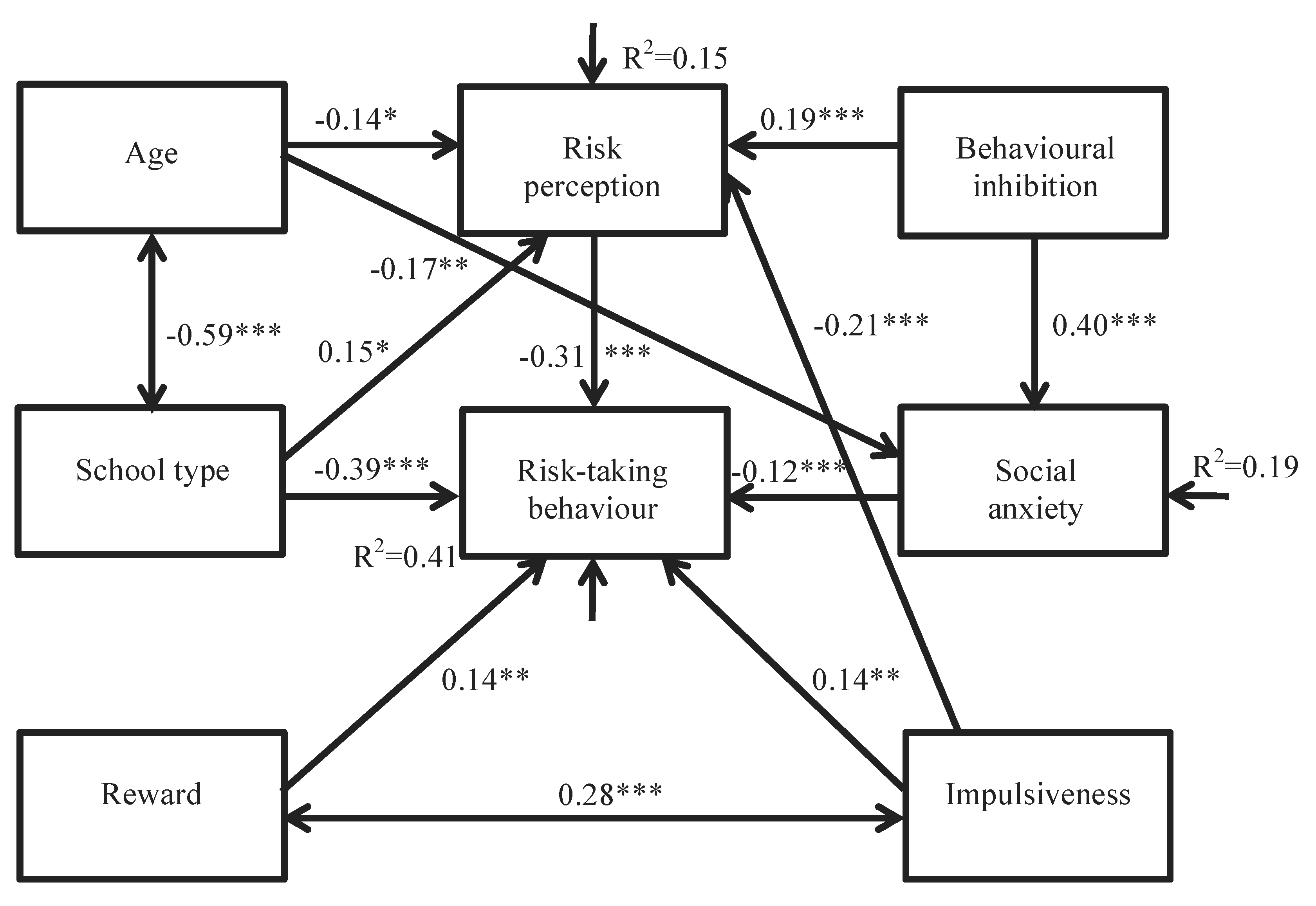

Supplement: S3 Fig — * p<0.05; ** p<0.01; *** p<0.001. Boxes represent observed variables. Long, solid arrows represent regressions. Short arrows represent residual error variances that indicate the variation left unexplained by the variables in the path model. Numbers indicate the standardised regression weights and R2 indicates the amount of variance explained by the model. (TIFF) [file pone.0153842.s003.tiff]
