# Supplementary material for: Risk Perception and Risk-Taking Behaviour during Adolescence: The Influence of Personality and Gender
Source: PLoS One. 2016 Apr 21;11(4):e0153842. doi: 10.1371/journal.pone.0153842 (PMC4839773; doi:10.1371/journal.pone.0153842)
Supplement: S1 Table — df = degrees of freedom, RMSEA = Root Mean Squared Error of Approximation, CI = confidence interval, CFI = Bentler’s Comparative Fit Index, TLI = Tucker-Lewis Index, SRMR = Standardised Root Mean Square Residual and AIC = Aikaike’s Information Criterion (DOCX) [file pone.0153842.s004.docx]

**S1 Table. Goodness of fit tests and indices.**

| **Model** | **Parameters** | **Goodness of fit measure** | |  |  |  |  |
| --- | --- | --- | --- | --- | --- | --- | --- |
|  | (estimated) | χ²(df), *p* | RMSEA (90% CI) | CFI | TLI | SRMR | AIC |
| **Model 4** | 36 (21) | χ²(15)=20.120, *p*=0.167 | 0.035 (0.000-0.072) | 0.987 | 0.975 | 0.0427 | 62.120 |

df = degrees of freedom, RMSEA = Root Mean Squared Error of Approximation, CI = confidence interval, CFI = Bentler’s Comparative Fit Index, TLI = Tucker-Lewis Index, SRMR = Standardised Root Mean Square Residual and AIC = Aikaike’s Information Criterion
